# General two-level framework for demand-responsive transport optimization

**DOI:** 10.1038/s41598-026-44833-6

**Published:** 2026-03-22

**Authors:** Andrzej Bozek, Tomasz Krzeszowski, Tomasz Sliwa

**Affiliations:** Faculty of Electrical and Computer Engineering, Rzeszow University of Technology, al. Powstancow Warszawy 12, Rzeszow, 35-959 Poland

**Keywords:** Demand-responsive transport, Smart city, Constraint programming, Vehicle routing, Electric vehicle, Tardiness minimization, Energy science and technology, Engineering, Mathematics and computing

## Abstract

This paper proposes a generalized optimization framework for demand-responsive transport (DRT), a modern form of transport organization in which services are planned and provided directly in response to the client demand. The developed framework has a two-level structure. The first level includes basic features essential to every DRT system. The second level takes into account the electric vehicle energy consumption and charging, predefined initial parts of vehicle routes for combining consecutive vehicle planning windows, as well as special passenger requests, such as a dedicated space for disabled people or Wi-Fi network availability. Three variants of models have been prepared, differing in how vehicles move between bus stops: freely point-to-point planning (General), restricted sections of the vehicle path (Sections) and fixed routes of every vehicle (Routes). During experiments, the developed model was thoroughly tested, in particular, its effectiveness in the basic version and with additional extensions was evaluated. The tests were performed on a dataset created based on the real public transport system of Rzeszow, Poland, from which 64 bus stops were mapped. The optimization process involved 200 passengers. The experiments confirmed the usefulness of the proposed solution.

## Introduction

Demand-responsive transport (DRT) is a modern form of transport organization in which services are planned and provided directly in response to the demand of the clients. The most characteristic features of DRT systems include: booking and control of transport supported by modern devices and IT systems^1^, e.g. mobile applications, automatic assignment of passengers to vehicles, departure time determining, and flexible vehicle routing in ad hoc manner based on optimization algorithms^2–4^. The domain of DRT systems is very extensive and is still developing rapidly. For this reason, there are many systems and optimization problems that are identical or similar to DRT, but named differently, e.g. a demand responsive shared transport (DRST)^5^, dial-a-ride problem (DARP)^6^, dial-a-ride service^7^, demand-responsive customized bus^8,9^, customized bus routing problem (CBRP)^10^, customized bus scheduling problem^11^. Mobility as a Service (MaaS) is a concept similar to DRT, with an emphasis on multimodal transport and the use of modern digital tools for the integration of services, such as planning, booking, and payment of transportation by smartphone^12^.

The popularity of DRT has become global and has spread to various continents, regions, countries, and cities. Many studies have examined the feasibility and benefits of DRT, both in planned and existing implementations. For instance, such research includes case studies in Amsterdam (Netherlands)^13^, Bremerhaven (Germany)^14^, Lolland (Denmark)^15^, Ragusa (Italy)^5^, Luxembourg^7^, Valencia (Spain)^16^, Velenje (Slovenia)^17^, northeastern Hungary^18^, Bristol (UK)^1^, Sao Paulo (Brazil)^19^, Beijing (China)^10^, Chaoyang District of Beijing (China)^20^, Chengdu (China)^3,11^. These analyzes are often applied to the suburbs and rural areas^13–18^, which are areas expected to benefit the most from the use of DRT.

A number of works cover analysis and predictions about the expected features and benefits of DRT in a given place and scenario, e.g., replacing the regular bus service and fixed routes by DRT^13,15,18,21^, combining regular transit with DRT^22^, using electric vehicles in DRT systems^7,23^, passenger transfers between vehicles^24^, using flexible dynamically determined routes^2^, combining DRT with an electric bike-sharing system^17^, comparison of taxi vs. DRST systems^5^, DRT systems for first/last mile feeder transportation^1,19^, feeding metro stations by DRT^4^, DRT-based connectors between residential areas and transit hubs^25^, DRT supported by priorities at signalized intersections^26^. There are also works that provide generalized methods and frameworks for analyzing DRT systems at strategic, tactical, and operational levels^27^, and take into account policy, regulation, funding, technologies, interactions of travel demand with operation parameters, as well as the impacts of DRT on mobility, society, and the environment^28^. Research on autonomous vehicles (AVs) and adaptive cruise control (ACC)^29,30^ is also important in the context of DRT, as such vehicles are well suited to planned on-demand transport.

DRT involves problems of data collecting and management, IT system integration, mobile and responsive application development, and, in particular, optimization. There are extensive reviews that consider DRT mainly from an optimization point of view^6,27,31–33^. Some of them focus on selected aspects, such as charging management in electrified DRT^23^, or specific countries, such as Great Britain^31^.

In terms of responsiveness, DRT optimization algorithms can generally be divided into static and dynamic^6^. A more detailed classification distinguishes three groups of algorithms: dynamic online – providing the highest responsiveness for booking (up to “last minute”) and also on unexpected disturbances, dynamic offline – only allowing changes of transits that have not already started, and static – meaning that all planning takes place before all the service is launched^9^. The certainty of the information provided for optimization is the second important aspect of classification. Hence, planning is called deterministic in the case of reliable and stable data and stochastic otherwise^6^.

Planning the operation of a DRT system is a difficult optimization task, both due to the significant structural complexity of such a system and the high computational complexity. For this reason, many dedicated optimization algorithms have been developed, tested, and compared with each other. Sometimes exact algorithms are used^33^: branch-and-bound, branch-and-cut, branch-and-price, gradient descent, mixed integer linear programming^11^. However, for the real-world size cases, heuristics and metaheuristics are often much more appropriate, namely^33^: genetic algorithm, particle swarm optimization, variable neighborhood search, large neighborhood search, local search, tabu search, simulated annealing. Simulation methods are also used quite often^15,19^, especially those based on agent systems^1,5,14,21^. The objectives of optimization in DRT systems include: time and distance, ridership, population’s perception^2,7,13,20,25^, greenhouse gas emission per kilometer, especially CO2^13,15,20^, general savings, and operator’s revenue^4,7,11,14,24,34^.

Research has shown many properties and advantages of DRT systems. In rural areas, DRT can replace regular bus transport, leading to a larger number of smaller vehicles, resulting in a similar total cost with reduced CO2 emissions^15^. Other studies have shown that a DRT system can provide economic and environmental savings when connecting urban and rural areas^14^. According to the simulation result^5^, a DRT system may outperform taxis in the case of high enough demand rates, but it is relatively less effective for smaller rates. A demand-responsive system connecting residential areas and transit hubs ensures cost advantages, especially in the scenario of frequent transits and small distances between the hubs^25^. Extending a DRT optimization algorithm to schedule passenger transfers resulted in an additional 8% savings on real-life data instances^24^. It was also confirmed that a non-fixed stop DRT system may reduce total travel time by up to about 9% compared to fixed stop service^20^.

This work is motivated by the problem of preparing a prototype DRT optimization module dedicated for Rzeszow, Poland. The project specified various assumptions. First of all, the system should support about 60 bus stops, vehicle waiting times at bus stops are limited, the earliest presence time for each passenger is given, and travel between each pair of bus stops must be possible (with transfers, if needed). Moreover, the system must support continuous, interval-wise travel scheduling, i.e., each subsequent scheduling interval needs to be compatible with the previous one, so that the vehicle routes planned in the previous interval are executed in the next one and the assigned passengers are transported while also planning routes for new passengers. It is also assumed that some of the vehicles are electric, and the entire charging planning process must be taken into account. Finally, the system has to match vehicles to passengers according to their specific requirements, such as Wi-Fi access, bicycle transport, etc.

Although the specified optimization assumptions seem typical and obvious, their required combination cannot be found in existing works. Existing studies either analyze the suitability of DRT under given conditions without implementing a specific scheduling mechanism, or rely on traffic simulations without providing any mechanisms for scheduling individual passenger requests, or abstractly concern quite specific aspects of DRT optimization (e.g., time windows^10^, transfers^24^, and electric vehicles^7,23^). None of these approaches leads to a general optimization system that includes even most of the functions listed in our specification.

We decided to avoid developing a closed system for the precisely defined specification that would then be unusable for other, even similar, problems. Instead, we developed a general framework for optimization with extensive customization and adaptability. There are three aspects of this generalization:

1. The framework has a two-level structure. The first level involves basic features needed in virtually every DRT system, i.e. vehicle-passenger assignment and vehicle route planning. On the second level, all additional functions are embedded, e.g., continuous interval-wise travel scheduling, management of electric vehicle charging, etc. A user is free to utilize the framework as it is, if its features are appropriate, may employ only the first layer in the case of simpler scenarios, may develop a custom second level for specific requirements, or, finally, both levels can be slightly adapted to a new specification.
2. There are three routing options proposed to choose, from *General* point-to-point connections, through combining *Sections* of predefined lines, up to assignment vehicles to fixed *Routes*. This order imposes decreasing flexibility but also decreasing computational complexity, so the best choice will depend on the specific problem type, the problem size, and the optimization tools used.
3. The framework is formulated in the mixed-integer linear programming (MILP) convention, which leaves a wide range of options for choosing an optimization approach and tool. One can use state-of-the-art MILP solver or implement a custom optimization algorithm (e.g. one based on a metaheuristic method).

Although the generalized optimization framework is proposed, its usefulness is presented in a specific example. The example involves 64 bus stops really existing in the Rzeszow public transport system, 6 vehicles (including 3 electric) having from 5 to 11 seats, and 200 passenger orders. Computational experiments were performed for the first level (*basic*) variant and for the (*extended*) case combining the two framework levels. In both cases, all three proposed routing schemes were compared, namely *General*, *Section*, and *Routes*. The IBM Constraint Programming Optimizer (CPO) solver^35^ was used for the computations.

## Problem formulation

The two-level DRT optimization problem is defined as follows:

1. The planning involves *P* passengers, *V* vehicles, and *B* bus stops. The numbers in the sets $\mathcal {P}=\{1,2,\ldots ,P\}$, $\mathcal {V}=\{1,2,\ldots ,V\}$, and $\mathcal {B}=\{1,2,\ldots ,B\}$ represent the identifiers of the passengers, vehicles, and bus stops, respectively.
2. For each ordered pair of bus stops $(x,y)\in \mathcal {B}^2$ are defined:

- travel distance $$D_{(x,y)}$$,
- travel time $$T_{(x,y)}$$.

3. For each bus stop $b\in \mathcal {B}$ a maximum waiting time $W_b$ is defined. A vehicle is not allowed to remain at the bus stop for a time longer than $W_b$.
4. The number of seats $s_v$, i.e., the maximum number of passengers carried, is defined for each vehicle $v\in \mathcal {V}$.
5. For each passenger $p\in \mathcal {P}$, the following are defined:

- a start bus stop $x_p\in \mathcal {B}$;
- an end bus stop $y_p\in \mathcal {B}$;
- the earliest time $e_p$ at which the passenger can appear at the start bus stop;
- the latest time $l_p$ at which the passenger wants to reach the end bus stop.

6. A set of *K* template routes is defined. Each one has the form $R_k=\left( r^k_1,r^k_2,\ldots ,r^k_{g_k},r^k_1,\ldots \right)$, $r^k_i\in \mathcal {B}$, $k\in \{1,2,\ldots ,K\}$ of a cyclic sequence of bus stops. The set of template routes is constructed in such a way that the directed graph
  1 $$\begin{aligned} \textbf{G}=(\textbf{N},\textbf{A}),\quad \textbf{N}=\bigcup \limits _{k=1}^K\bigcup \limits _{i=1}^{g_k}\{r^k_i\},\quad \textbf{A}=\bigcup \limits _{k=1}^K\left[ \left\{ (r^k_{g_k},r^k_1)\right\} \cup \bigcup \limits _{i=1}^{g_k-1}\left\{ (r^k_i,r^k_{i+1})\right\} \right] \end{aligned}$$
  has a path from $x_p$ to $y_p$ for each $p\in \mathcal {P}$.
7. For each vehicle $v\in \mathcal {V}$ an initial schedule can be defined. It represents a part of the route established prior to the execution of the algorithm that must be accomplished before new elements of the route are added. The initial schedule is characterized by the following items:

- a number of bus stops included in the initial schedule $z_v\in \{0\}\cup \mathbb {N}$, if $z_v=0$, then there is no initial schedule for the vehicle *v*;
- an identifier $^*b^v_i\in \mathcal {B}$ of the *i*-th bus stop for $i\in \{1,2,\ldots ,z_v\}$;
- an arrival time $^*a^v_i$ at the *i*-th bus stop for $i\in \{1,2,\ldots ,z_v\}$;
- a departure time $^*d^v_i$ at the *i*-th bus stop for $i\in \{1,2,\ldots ,z_v\!-\!1\}$, the departure time from *z*-th is not included in the initial plan, as determined by the algorithm;
- a number $^*s^v_i$ of the seats occupied by passengers from the initial schedule between the *i*-th and $(i\!+\!1)$-th bus stop for $i\in \{1,2,\ldots ,z_v\!-\!1\}$.

It is still possible to add new passengers to the route segments starting and/or ending at stops $i\le z_v$, if times ${^*a^v_i}$, ${^*d^v_i}$ are suitable for them, but the number of such passengers cannot exceed $s_v-{^*s^v_i}$. The times ${^*a^v_i}$ and ${^*d^v_i}$ can be arbitrary (and should be reasonable) and are not subject to general restrictions that involve travel times, waiting times, and energy consumption. This is due to the assumption that the initial schedule may be based on different parameters.

8. The assignment of passengers to vehicles and bus stops is done in the same way whether a bus stop belongs to the initial schedule or to the new part of the schedule. The only difference is that the number of free seats in a vehicle $v\in V$ is decreased by $^*s^v_i$ for travel between pairs of bus stops $(i, i\!+\!1)$, where $i\in \{1,2,\ldots ,z_v\!-\!1\}$.
9. For each bus stop $b\in \mathcal {B}$ the installed charger power $E^\textrm{P}_b$ is defined. If $E^\textrm{P}_b=0$, there is no electric vehicle charger at the bus stop *b*.
10. For each vehicle $v\in V$ parameters related to electric drive are defined:

- coefficient of energy consumption per unit of distance $E^\textrm{e}_v$;
- vehicle battery capacity $E^\textrm{c}_v$;
- initial energy of the battery $E^0_v$, given at the moment of arrival at the bus stop with the sequence number $\max \{1,z_v\}$.

If the vehicle has no electric drive, then $E^\textrm{e}_v=E^\textrm{c}_v=E^0_v=0$.

11. The energy of a vehicle’s battery increases by $tE^\textrm{P}_b$ when charging for time *t* at the bus stop $b\in \mathcal {B}$. The battery energy decreases by $dE^\textrm{e}_v$ when a vehicle $v\in \mathcal {V}$ travels the distance *d*. A feasible solution to the problem has to ensure that the battery energy of each vehicle $v\in \mathcal {V}$ remains in the interval $[0,E^\textrm{c}_v]$ at any moment of the schedule.
12. A set *F* of special features is defined.
13. A subset $f_v\subseteq F$ (possibly empty) is defined for each vehicle $v\in \mathcal {V}$ that represents the special features provided by the vehicle.
14. A subset $u_p\subseteq F$ (possibly empty) is defined for each passenger $p\in \mathcal {P}$ that represents the special features requested by that passenger.
15. A passenger $p\in \mathcal {P}$ can be assigned to a vehicle $v\in \mathcal {V}$ if the vehicle meets the passenger’s requirements, i.e., if $u_p\subseteq f_v$.

Items up to 6 specify the basic level, while the items that follow it constitute the extension.

The solution of problem $\mathcal {S}=(\mathcal {Q},\mathcal {R})$ consists of:

1. The set
  2 $$\begin{aligned} \mathcal {Q}=\left\{ \kappa _p\,|\,p\in \mathcal {P}\right\} ,\qquad \kappa _p=\left( (\gamma ^p_1,\eta ^p_1,\mu ^p_1),(\gamma ^p_2,\eta ^p_2,\mu ^p_2),\ldots (\gamma ^p_{q_p},\eta ^p_{q_p},\mu ^p_{q_p})\right) \end{aligned}$$
  of sequences describing the assignment of passengers to vehicles and bus stops. A sequence $\kappa _p$ of $q_p$ triples defines the assignment for the passenger $p\in \mathcal {P}$, where the triple $\left( \gamma ^p_k,\eta ^p_k,\mu ^p_k\right)$ describes the *k*-th part of the passenger travel in the assigned vehicle $\gamma ^p_k\in \mathcal {V}$. The values $\eta ^p_k, \mu ^p_k$ represent the sequence numbers of the passenger entry and exit bus stops on the vehicle route, respectively. The passenger *p* has $q_p\!-\!1$ transfers between buses. In particular, $\kappa _p=\left( (\gamma ^p_1,\eta ^p_1,\mu ^p_1)\right)$ in the case of travel without any transfer.
2. The set
  3 $$\begin{aligned} \mathcal {R}=\left\{ \rho _v\,|\,v\in \mathcal {V}\right\} ,\qquad \rho _v=\left( (\beta ^v_1,\overline{a}^v_1,\overline{d}^v_1),(\beta ^v_2,\overline{a}^v_2,\overline{d}^v_2),\ldots ,(\beta ^v_{h_v},\overline{a}^v_{h_v},\overline{d}^v_{h_v})\right) \end{aligned}$$
  representing planned routes for vehicles. The route of the vehicle $v\in \mathcal {V}$ is represented by a sequence $\rho _v$ of $h_v$ triples, where the triple $\left( \beta ^v_i,\overline{a}^v_i,\overline{d}^v_i\right)$ describes the *i*-th bus stop visited by the vehicle, specifying the stop’s number $\beta ^v_i\in \mathcal {B}$, the arrival time $\overline{a}^v_i$, and the departure time $\overline{d}^v_i$.

The objective is to find a solution $\mathcal {S}$ that minimizes the function

4 $$\begin{aligned} T_\textrm{avg}(\mathcal {S})=\frac{1}{P}\sum \limits _{p\in \mathcal {P}}\max \left( \overline{a}^{\gamma ^p_{q_p}}_{\mu ^p_{q_p}}-l_p,0\right) , \end{aligned}$$

representing the average tardiness of all passengers.

In computational experiments, the primary objective $T_\textrm{avg}(\mathcal {S})$ will also be compared with some others, namely

5a $$\begin{aligned} T_\textrm{max}(\mathcal {S})=\max \limits _{p\in \mathcal {P}}\left\{ \max \left( \overline{a}^{\gamma ^p_{q_p}}_{\mu ^p_{q_p}}-l_p,0\right) \right\} , \end{aligned}$$

5b $$\begin{aligned} U_\textrm{sum}(\mathcal {S})=\sum \limits _{p\in \mathcal {P}, \; \; \overline{a}^{\gamma ^p_{q_p}}_{\mu ^p_{q_p}}>l_p}1, \end{aligned}$$

5c $$\begin{aligned} A_\textrm{avg}(\mathcal {S})=\frac{1}{P}\sum \limits _{p\in \mathcal {P}}\overline{a}^{\gamma ^p_{q_p}}_{\mu ^p_{q_p}}, \end{aligned}$$

providing the maximum passenger tardiness, the number of tardy passengers, and the average passenger arrival time, respectively.

### Levels of routing flexibility

**Fig. 1:**
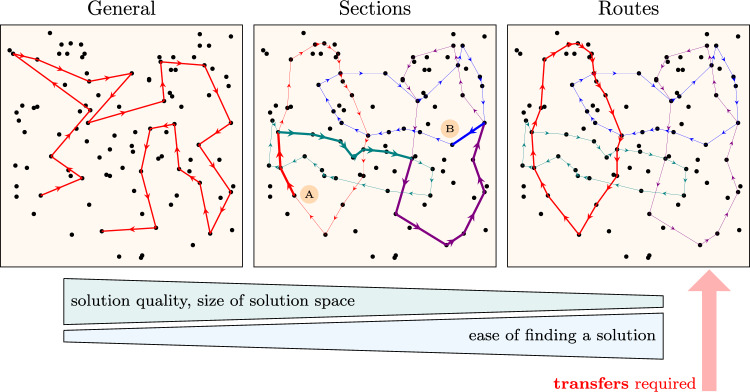
Three levels of routing flexibility.

The three levels of vehicle routing flexibility will be considered, as illustrated in Fig. 1. They can be specified as follows:

- *General* – this level has no restrictions on the selection of route segments between bus stops. A vehicle can move directly from any stop $b_i\in \mathcal {B}$ to any other $b_j\in \mathcal {B}$;
- *Sections* – vehicles can move only along sections of predefined routes $R_k$ (see problem formulation item 6), in other words, along arcs of the graph $\textbf{G}$ (1). However, any output arc can be chosen at route crossovers. For example, if a passenger needs to travel from point A to B (Fig. 1), an assigned vehicle can first move along a part of the red route, then green and violet, and finally blue one;
- *Routes* – each vehicle has to move circularly along one predefined route $R_k$, which is chosen for the entire schedule, and the vehicle starts from the first stop of the route (bus depot).

The level *General* induces the largest solution space and thus potentially includes better solutions than the other levels, but these can be harder to find due to computational complexity. The opposite situation occurs in the case of the level *Routes*. Therefore, it is not known in advance which level may be the best for a specific solver and problem data, so all three will be experimentally tested.

In the case of the level *Routes*, passenger transfers are required, as individual routes do not reach every bus stop. For this reason, we introduce the sequences

6 $$\begin{aligned} \Gamma _p=\left( c^p_1,c^p_2,\ldots ,c^p_{q_p-1}\right) ,\quad c^p_k\in \mathcal {B}, \qquad \forall \;p\in \mathcal {P},\,k\in \left\{ 1,2,\ldots ,q_p\!-\!1\right\} \end{aligned}$$

specifying the intermediate (transfer) bus stops $c^p_1,c^p_2,\ldots ,c^p_{q_p-1}$ for the passenger *p* between the endpoints $x_p$ and $y_p$, so that each segment between consecutive stops can be traveled in one vehicle. The parameter $q_p$ represents the number of travel segments of the passenger *p* and, in particular, the travel has no transfers if $q_p=1$. The sequences $\Gamma _p$ are not considered independent problem data, because they can be derived from $D_{(x,y)}$, $T_{(x,y)}$, $R_k$ based on various assumptions (minimum number of transfers, minimum distance, and minimum travel time excluding waiting at stops, etc.).

## Constraint programming model

### Basic variant

All decision variables are denoted using Greek letters to easily distinguish them from other symbols. The basic level model uses the following decision variables:

7a $$\begin{aligned}&\beta ^v_i\in \mathcal {B}, & \forall \;v\in \mathcal {V},\,i\in \left\{ 1,2,\ldots ,S\right\} ,\end{aligned}$$

7b $$\begin{aligned}&\psi _v\in \left[ 0,H\right] , & \forall \;v\in \mathcal {V},\end{aligned}$$

7c $$\begin{aligned}&\omega ^v_i\in \left[ 0,W\right] , & \forall \;v\in \mathcal {V},\,i\in \left\{ 1,2,\ldots ,S\right\} ,\end{aligned}$$

7d $$\begin{aligned}&\tau ^v_i\in \left[ 0,D\right] , & \forall \;v\in \mathcal {V},\,i\in \left\{ 1,2,\ldots ,S\!-\!1\right\} ,\end{aligned}$$

7e $$\begin{aligned}&\gamma ^p_k\in \mathcal {V}, & \forall \;p\in \mathcal {P},\,k\in \left\{ 1,2,\ldots ,q_p\right\} ,\end{aligned}$$

7f $$\begin{aligned}&\eta ^p_k\in \left\{ 1,2,\ldots ,S\right\} , & \forall \;p\in \mathcal {P},\,k\in \left\{ 1,2,\ldots ,q_p\right\} ,\end{aligned}$$

7g $$\begin{aligned}&\mu ^p_k\in \left\{ 1,2,\ldots ,S\right\} , & \forall \;p\in \mathcal {P},\,k\in \left\{ 1,2,\ldots ,q_p\right\} ,\end{aligned}$$

7h $$\begin{aligned}&\delta _v\in \left\{ 1,2,\ldots ,K\right\} , & \forall \;v\in \mathcal {V}. \end{aligned}$$

These variables have the following meaning:

- $\beta ^v_i$ – the *i*-th bus stop of the vehicle *v* route;
- $\psi _v$ – the time at which the vehicle *v* arrives at the first bus stop;
- $\omega ^v_i$ – waiting time of the vehicle *v* at the *i*-th bus stop of its route;
- $\tau ^v_i$ – the travel time between the *i*-th and $(i\!+\!1)$-th bus stops of the vehicle *v* route;
- $\gamma ^p_k$ – the vehicle assigned to the passenger *p* for the *k*-th segment of his travel;
- $\eta ^p_k$, $\mu ^p_k$ – the sequence number of the vehicle $\gamma ^p_k$ bus stop at which the passenger *p* starts and finishes, respectively, the *k*-th segment of his travel;
- $\delta _v$ – the number of predefined routes assigned to the vehicle *v* (applicable only in the *Routes* variant).

The constants *W* and *D* bound the respective variable domains from above and are defined as the following maximums

8a $$\begin{aligned} W=\max \limits _{b\in \mathcal {B}}W_b, \end{aligned}$$

8b $$\begin{aligned} D=\max \limits _{(x,y)\in \mathcal {B}^2}D_{(x,y)}. \end{aligned}$$

The constant *H* denotes the maximum allowable time for a vehicle to reach the initial stop along its designated route.

For convenience, a few decision expressions are defined that simplify the formulation of constraints. All decision expressions are marked by overlines:

9a $$\begin{aligned}&\overline{a}^v_i\;:=\; {\left\{ \begin{array}{ll} \psi _v,& \text {if}\quad i=0,\\ \overline{d}^v_{i-1}+\tau ^v_{i-1},& \text {otherwise}, \end{array}\right. } & \forall \;v\in \mathcal {V},\,i\in \left\{ 1,2,\ldots ,S\right\} ,\end{aligned}$$

9b $$\begin{aligned}&\overline{d}^v_i\;:=\;\overline{a}^v_i+\omega ^v_i, & \forall \;v\in \mathcal {V},\,i\in \left\{ 1,2,\ldots ,S\!-\!1\right\} , \end{aligned}$$

10 $$\begin{aligned}&\{0,1\}\ni {^p_k\overline{o}^v_i}\;:=\;\left( \gamma ^p_k=v\right) \wedge \left( \eta ^p_k\le i\le \mu ^p_k-1\right) , & \begin{aligned}\forall \;&p\in \mathcal {P},\,k\in \left\{ 1,2,\ldots ,q_p\right\} ,\\&v\in \mathcal {V},\,i\in \left\{ 1,2,\ldots ,S\!-\!1\right\} .\end{aligned} \end{aligned}$$

The expressions $\overline{a}^v_i$ and $\overline{d}^v_i$ represent the arrival/departure time of the vehicle *v* at/from the *i*-th stop of its route, respectively. They depend on the decision variables $\psi _v$, $\tau ^v_i$ and $\omega ^v_i$, according to (9a)-(9b). The binary expression ${^p_k\overline{o}^v_i}$ in (10) takes the value 1 if and only if the passenger *p* in the *k*-th part of its travel occupies a seat in vehicle *v* during the *i*-th stage of its route.

The following constraints are defined for the model:

11a $$\begin{aligned}&{General:} & \beta ^v_i\ne \beta ^v_{i+1}, & \forall \;v\in \mathcal {V},\,i\in \left\{ 1,2,\ldots ,S\!-\!1\right\} ,\end{aligned}$$

11b $$\begin{aligned}&{Sections:} & \left( \beta ^v_i,\beta ^v_{i+1}\right) \in \textbf{A}, & \forall \;v\in \mathcal {V},\,i\in \left\{ 1,2,\ldots ,S\!-\!1\right\} ,\end{aligned}$$

11c $$\begin{aligned}&{Routes:} & \left( \beta ^v_i,\beta ^v_{i+1}\right) =\left( r^{\delta _v}_{\iota _1},r^{\delta _v}_{\iota _2}\right) , & \begin{aligned}\forall \;&v\in \mathcal {V},\,i\in \left\{ 1,2,\ldots ,S\!-\!1\right\} ,\\&\iota _1=1+\left( (i\!-\!1)\mod g_{\delta _v}\right) ,\\&\iota _2=1+\left( i\mod g_{\delta _v}\right) ,\end{aligned} \end{aligned}$$

12 $$\begin{aligned}&\eta ^p_k<\mu ^p_k, & \forall \;p\in \mathcal {P},\,k\in \left\{ 1,2,\ldots ,q_p\right\} , \end{aligned}$$

13a $$\begin{aligned}&\beta ^{\gamma ^p_1}_{\eta ^p_1}=x_p,\quad \beta ^{\gamma ^p_{q_p}}_{\mu ^p_{q_p}}=y_p, & \forall \;p\in \mathcal {P},\end{aligned}$$

13b $$\begin{aligned}&\beta ^{\gamma ^p_{q_p}}_{\eta ^t_{q_p}}=c^p_{q_p-1},\quad \beta ^{\gamma ^p_1}_{\mu ^p_1}=c^p_1, & \forall \;p\in \mathcal {P},\,q_p>1,\end{aligned}$$

13c $$\begin{aligned}&\beta ^{\gamma ^p_{k+1}}_{\eta ^p_{k+1}}=c^p_k,\quad \beta ^{\gamma ^p_{k+1}}_{\mu ^p_{k+1}}=c^p_{k+1}, & \forall \;p\in \mathcal {P},\,k\in \{1,2,\ldots ,q_p\!-\!2\}, \end{aligned}$$

14a $$\begin{aligned}&\omega ^v_i\le W_{\beta ^v_i}, & \forall \;v\in \mathcal {V},\,i\in \left\{ 1,2,\ldots ,S\right\} ,\end{aligned}$$

14b $$\begin{aligned}&\tau ^v_i=T_{\left( \beta ^v_i,\beta ^v_{i+1}\right) }, & \forall \;v\in \mathcal {V},\,i\in \left\{ 1,2,\ldots ,S\!-\!1\right\} , \end{aligned}$$

15a $$\begin{aligned}&\overline{d}^{\gamma ^p_1}_{\eta ^p_1}\ge e_p, & \forall \;p\in \mathcal {P},\end{aligned}$$

15b $$\begin{aligned}&\overline{a}^{\gamma ^p_k}_{\mu ^p_k}\le \overline{d}^{\gamma ^p_{k+1}}_{\eta ^p_{k+1}}, & \forall \;p\in \mathcal {P},\,k\in \{1,2,\ldots ,q_p\!-\!1\}, \end{aligned}$$

16 $$\begin{aligned}&\sum \limits _{p\in \mathcal {P}}\sum \limits _{k=1}^{q_p}{^p_k\overline{o}^v_i}\le s_v, & \forall \;v\in \mathcal {V},\,i\in \left\{ 1,2,\ldots ,S\!-\!1\right\} . \end{aligned}$$

The constraints (11a)-(11c) control the route order and depend on the selected level of routing flexibility. For the level *Sections*, only the route segments from the set $\textbf{A}$ are allowed (11b). In the case of the level *Routes*, the predefined routes $R_k$ chosen by the decision variables $\delta _v$ are cyclically performed by the vehicles (11c). The condition $\beta ^v_i\ne \beta ^v_{i+1}$ is always enforced, directly for the level *General* (11a) and indirectly for the others. It is necessary to properly apply $W_b$ as the maximum waiting time of the vehicle at the bus stop *b*. Without this condition, a vehicle could stay at the same stop for many stages of the route, accumulating a waiting time at the same bus stop multiple times.

The constraints (12) assert that for every passenger *p*, the initial bus stop precedes the final one on each *k*-th travel part.

The constraints (13a)-(13c) correlate vehicle routes and passenger bus stops. If a travel has no transfer, i.e. $q_p=1$, then only (13a) is relevant and states that the bus stop of the vehicle assigned to the passenger *p* departure is consistent with the travel start, namely $\beta ^{\gamma ^p_1}_{\eta ^p_1}=x_p$, and similarly for the final point of the travel. If $q_p=2$, then (13b) extends the conditions to the transfer point $c^p_1$, while (13c) extends them further when $q_p>2$.

The time relations are introduced in the constraints (14a)-(15b). In the context of a vehicle, the waiting time at a bus stop cannot exceed the predefined limit (14a) and the travel time of a route segment is determined by its endpoints (14b). From the perspective of a passenger, the departure from the first bus stop of their travel cannot be faster than the earliest presence time $e_p$ (15a), and also any travel part has to finish before the start of the next one in the case of transfers (15b).

The left-hand side of (16) represents the total number of seats occupied on the *i*-th route segment of the vehicle *v* by all passengers at all parts of their travels. Therefore, this expression needs to be bounded from above by the number of available seats $s_v$.

### Extended variant

The extended DRT model includes additional features specified in Section Problem formulation starting with item 7. There is one more group of decision variables in the extended model

17 $$\begin{aligned}&\varepsilon ^v_i\in \left[ 0,W\right] , & \forall \;v\in \mathcal {V},\,i\in \left\{ 1,2,\ldots ,S\right\} , \end{aligned}$$

where $\varepsilon ^v_i$ represents the charging time of the vehicle *v* at the *i*-th bus stop on its route.

For brevity, the parameters

18 $$\begin{aligned} i_v=\max \{1,z_v\},\qquad \forall \;v\in \mathcal {V} \end{aligned}$$

are used in subsequent formulas. Only modified or new model elements are provided below. The ones that remain unchanged from the basic model are not repeated.

In the extended model, the following new or changed decision expressions are used:

19a $$\begin{aligned}&\overline{a}^v_i\;:=\; {\left\{ \begin{array}{ll} {^*a}^v_i,& \text {if}\quad i\le z_v>0,\\ \psi _v,& \text {if}\quad i=z_v=0,\\ \overline{d}^v_{i-1}+\tau ^v_{i-1},& \text {otherwise}, \end{array}\right. } & \forall \;i\in \left\{ 1,2,\ldots ,S\right\} ,\,v\in \mathcal {V},\end{aligned}$$

19b $$\begin{aligned}&\overline{d}^v_i\;:=\; {\left\{ \begin{array}{ll} {^*d}^v_i,& \text {if}\quad i<z_v,\\ \overline{a}^v_i+\omega ^v_i,& \text {otherwise}, \end{array}\right. } & \forall \;i\in \left\{ 1,2,\ldots ,S\!-\!1\right\} ,\,v\in \mathcal {V}, \end{aligned}$$

20a20b The expressions (19a)-(19b) are counterparts of (9a)-(9b) in the basic model, but the values $\overline{a}^v_i$ and $\overline{d}^v_i$ are fixed at ${^*a}^v_i$ and ${^*d}^v_i$, respectively, for the bus stops specified in the initial schedule (see item 7 of the problem formulation in Section Problem formulation).

The expressions (10) are still used in the extended model because the method of determining seat occupancy remains the same. However, it is worth highlighting that ${^p_k\overline{o}^v_i}$ represents the number of seats assigned to new passengers. The number of seats reserved in the initial schedule is given by $^*s^v_i$ (Section Problem formulation, item 7).

The new decision expressions (20a)-(20b) relate to the energy balance of electric vehicles. The expression $\overline{e}^v_i$ (20a) represents the battery energy of the vehicle *v* at the moment of arrival at the *i*-th bus stop. It is calculated as the energy at the departure from the previous stop reduced by consumption during travel. The expression  (20b), in turn, represents the battery energy of the vehicle *v* at the moment of departure from the *i*-th bus stop. It is the energy at arrival increased by charging while the vehicle is stationary.

The new or modified constraints of the extended model are given below.

21 $$\begin{aligned}&u_p\subseteq f_v, & \forall \;v\in \mathcal {V}, \end{aligned}$$

22 $$\begin{aligned}&\beta ^v_i={^*b^v_i}, & \forall \;v\in \mathcal {V},\,i\in \{1,2,\ldots ,z_v\}, \end{aligned}$$

23a $$\begin{aligned}&\textit{General:} & \beta ^v_i\ne \beta ^v_{i+1}, & \forall \;v\in \mathcal {V},\,i\in \left\{ i_v,i_v\!+\!1,\ldots ,S\!-\!1\right\} ,\end{aligned}$$

23b $$\begin{aligned}&\textit{Sections:} & \left( \beta ^v_i,\beta ^v_{i+1}\right) \in \textbf{A}, & \forall \;v\in \mathcal {V},\,i\in \left\{ i_v,i_v\!+\!1,\ldots ,S\!-\!1\right\} ,\end{aligned}$$

23c $$\begin{aligned}&\textit{Routes:} & \left( \beta ^v_i,\beta ^v_{i+1}\right) =\left( r^{\delta _v}_{\iota _1},r^{\delta _v}_{\iota _2}\right) , & \begin{aligned}\forall \;&v\in \mathcal {V},\,i\in \left\{ z_v\!+\!1,z_v\!+\!2,\ldots ,S\!-\!1\right\} ,\\&\iota _1=1+\left( (i\!-\!z_v\!-\!1)\mod g_{\delta _v}\right) ,\\&\iota _2=1+\left( (i\!-\!z_v)\mod g_{\delta _v}\right) ,\end{aligned} \end{aligned}$$

24a $$\begin{aligned}&\omega ^v_i\le W_{\beta ^v_i}, & \forall \;v\in \mathcal {V},\,i\in \left\{ i_v,i_v\!+\!1,\ldots ,S\right\} ,\end{aligned}$$

24b $$\begin{aligned}&\tau ^v_i=T_{\left( \beta ^v_i,\beta ^v_{i+1}\right) }, & \forall \;v\in \mathcal {V},\,i\in \left\{ i_v,i_v\!+\!1,\ldots ,S\!-\!1\right\} , \end{aligned}$$

25 $$\begin{aligned}&\sum \limits _{p\in \mathcal {P}}\sum \limits _{k=1}^{q_p}{^p_k\overline{o}^v_i}\le {\left\{ \begin{array}{ll} s_v-{^*s^v_i},& \text {if}\quad i<z_v,\\ s_v,& \text {otherwise}, \end{array}\right. } & \forall \;v\in \mathcal {V},\,i\in \left\{ 1,2,\ldots ,S\!-\!1\right\} . \end{aligned}$$

26The constraints (21) are new in the extended model and represent vehicle-bus matching based on special features (Section Problem formulation, items 12–15). New are also the constraints (22) that fix bus stops $\beta ^v_i$ for the parts of the routes included in the initial schedule.

The constraints (23a)-(23c) that follow (11a)-(11c) constitute the basic variant, but control the bus stop sequences excluding the initial schedule. In the case of the flexibility levels *General* and *Sections*, the control starts from the last bus stop of the initial schedule and the route segment $(z_v,z_v\!+\!1)$ is restricted by these constraints. In the case of *Routes* flexibility level, the control starts from the segment $(z_v\!+\!1,z_v\!+\!2)$ to allow the vehicle on the segment $(z_v,z_v\!+\!1)$ to move an arbitrary bus stop $\beta ^v_{z_v}$ to the first stop $r^{\delta _v}_1$ of the new cyclic route.

In the extended variant, the constraints (12)-(13c) are repeated without any changes, as they represent the rules of vehicle-passenger assignment which remain identical in this variant.

In the case of the constraints (14a)-(14b) of the basic model, only the indexing range changes that result in (24a)-(24b), because the travel and waiting times of the initial schedule are not subject to restrictions. However, the rules for passenger departure and arrival times remain unchanged, both for the initial and continued schedule, and therefore constraints (15a)-(15b) are inherited in the extended variant without change.

The constraints (16) take the form (25) in the extended model. The only change is that the number of free seats is $s_v-{^*s^v_i}$ for the route segments included in the initial schedules.

The last group of constraints (26) governs the energy constraints for electric vehicles. The energy cannot drop below 0 on arrival, when it reaches local minimums, and also cannot exceed the battery capacity $E^\textrm{c}_v$ before departure, when it reaches local maximums after charging. The charging time cannot be longer than the waiting time of a vehicle at a bus stop. If a vehicle $v\in \mathcal {V}$ is not electric, these constraints are trivially satisfied due to $E^\textrm{e}_v=E^\textrm{c}_v=E^0_v=0$ (Section Problem formulation, item 10).

## Implementation of optimization model

### Implementation details

The optimization model (Section Constraint programming model) has been implemented using IBM CPO, a modern solver that supports the constraint programming technique.

In the problem model, arrays of decision variables indexed by the values of other variables are often used, even in a two-dimensional arrangement. A representative example is $\beta ^{\gamma ^p_{k+1}}_{\eta ^p_{k+1}}$ (13c). Recall that *p* and *k* are fixed integers, $\gamma ^p_{k+1}$ (a vehicle assigned to a passenger) and $\eta ^p_{k+1}$ (a sequential number of bus stops assigned to a passenger) are individual decision variables statically indexed by *p* and *k*, and finally $\beta ^{\gamma ^p_{k+1}}_{\eta ^p_{k+1}}$ is the decision variable dynamically indexed from a two-dimensional array by the pair of indexes $I^2_{p,q}=\left( \gamma ^p_{k+1},\eta ^p_{k+1}\right)$. Such dynamic indexing is widely supported by constraint programming solvers and usually provided by a function element(variable, index). The CPO also has such a function, but it supports only one-dimensional indexing, so a flattening of the two-dimensional array is necessary. In the considered example, $\gamma ^p_{k+1}\in \{1,2,\ldots ,V\}$ (7e) and $\eta ^p_{k+1}\in \{1,2,\ldots ,V\}$ (7f), so the indexing $I^2_{p,q}$ spans over the $V\times S$ array that can be flattened to a one-dimensional form by the linear mapping $I^1_{p,q}=S\left( \gamma ^p_{k+1}-1\right) +\eta ^p_{k+1}-1$ with indexes starting from 0, which is also required by the CPO. Assuming that the collections of decision variables $\beta ^v_i$, $\gamma ^p_k$ and $\eta ^p_k$ are programmatically represented by the arrays beta[0..V*S-1], gamma[0..P-1][0..qp-1] and eta[0..P-1][0..qp-1], respectively, the decision structure $\beta ^{\gamma ^p_{k+1}}_{\eta ^p_{k+1}}$ obtains the programmatic implementation

$$\texttt {element(beta, S*(gamma[p-1][k]-1)+eta[p-1][k]-1)},$$

based on the element function. All other cases of dynamic indexing have been implemented in a similar way. Apart from the indexing, all expressions included in the model are linear with respect to the decision variables and therefore obvious in implementation.

The user of the model needs to fix two constants that are used to define the domains of the decision variables. The value *H* specifies the latest time at which a vehicle can arrive at the first stop on its route. The constant *S* represents the number of consecutive bus stops on the planned route for each vehicle. These constants are not direct problem parameters, but should be inferred from them or adjusted experimentally. In particular; if *S* is too low, the model will be infeasible, as the routes will not be long enough to transport all passengers. If *S* is large enough to ensure feasibility, it may still be too small to obtain a good quality solution. On the other hand, an over-scaled *S* will unnecessarily increase the number of decision variables and may also degrade the performance of optimization.

### Data preparation

Constraint programming solvers usually support only discrete numerical decision variables. Therefore, to model real-world values related to DRT, an appropriate scaling of physical values to their numerical representation must be ensured. The basic SI units of distance (meter, m), time (second, s), energy (joule, J), and power (watt, W), together with the resulting power consumption unit (J/m) provide the required resolution and precision. They have been chosen to represent the optimization quantities, as shown in Table 1. In the last column, the typical ranges of these quantities are converted from practical units to model units to confirm that their resolution is high enough.

**Table 1:** Units of the modeled quantities.

Quantity		Model unit		Typical range
Symbol	Description	Symbol	Name	
$D_{(x,y)}$	travel distance	m	meter	
$T_{(x,y)}$, $\tau ^v_i$	travel time	s	second	1..60 min = 60..3600 s
$\psi _v$, ${^*a^v_i}$, ${^*d^v_i}$	arriv./dep. time	s	second	1..60 min = 60..3600 s
$W_b$, $\omega ^v_i$	waiting time	s	second	1..60 min = 60..3600 s
$\varepsilon ^v_i$	charging time	s	second	1..60 min = 60..3600 s
$E^\textrm{0}_v$	init. battery energy	J	joule	10..200 kWh = $(0.36\,..\,7.2)\!\cdot \!10^8$ J
$E^\textrm{c}_v$	battery capacity	J	joule	10..200 kWh =$(0.36\,..\,7.2)\!\cdot \!10^8$ J
$E^\textrm{P}_b$	charging power	W	watt	50..200 kW = $(0.5\,..\,2.0)\!\cdot \!10^5$ W
$E^\textrm{e}_v$	energy consumption	J/m	$\mathrm {\frac{joule}{meter}}$	20..80 $\mathrm {\frac{kWh}{100\ km}}$ = 720..2880 J/m

The sequences of transfer bus stops $\Gamma _p$ (6) needed for the *Routes* flexibility level have been derived for each passenger $p\in \mathcal {P}$ from the data $x_p$, $y_p$, $T_{(x,y)}$, $R_k$. For this purpose, a two-stage version of a standard traveling salesman problem (TSP) optimization procedure was employed. First, a path is sought that minimizes the number of transfers between $x_p$, $y_p$. If there are many solutions with the same objective, the fastest one is sought in the second stage based on the total travel time.

### Solution retrieving

The solution $\mathcal {S}$ defined in Section Problem formulation is entirely composed of the decision variables and the values of the decision expressions included in the optimization model. In particular, the resulting passenger-vehicle assignment (2) consists of the decision variables $\gamma ^p_k$ (7e), $\eta ^p_k$ (7f), $\mu ^p_k$ (7g), while the vehicle routes representation (3) is based on the variables $\beta ^v_i$ (7a) and the expressions $\overline{a}^v_i$ (9a), (19a), $\overline{d}^v_i$ (9b), (19b). The proposed optimization model builds length routes *S* for all vehicles, so $h_p=S$ for each $v\in \mathcal {V}$ in (3).

## Results

As part of the experiments, the developed model was thoroughly tested, in particular, the effectiveness of the model in the basic version (see Section Basic variant) and with additional extensions (see Section Extended variant) was tested, which took into account the energy consumption of electric vehicles and their charging, different initial states of vehicles, as well as special passenger requests (a place for disabled people, availability of Wi-Fi network in the vehicle, a vending machine with drinks, etc.). For each of the above variants, two sets of test data were prepared, containing reports of 200 passengers and assuming a time window in which passengers appear, of two hours. Passengers were transported by 6 vehicles, including 3 electric vehicles, between 64 bus stops grouped into 6 routes. The first of the datasets (Dataset I) was generated in such a way that for the defined number of vehicles, there was an ideal solution (one in which none of the passengers was late after reaching the given destination). The algorithms in this variant were tested to check whether the developed model could find the correct solution and obtain zero delays for all passengers. In the second dataset (Dataset II), passengers were generated completely randomly (starting stop, ending stop, and departure and arrival times). This variant is closer to reality, and in this case, there does not necessarily exist (or it is very difficult to find) a solution in which there are no passenger delays. The number of passengers (200) and the time at which the passengers arrive (two hours) were experimentally selected in the initial phase of the research. During preliminary experiments, the model was tested for datasets with different numbers of passengers, different numbers of stops, and a time window of 18 hours (from 6 a.m. to midnight). The tests carried out showed that the model can handle the number of passengers up to about 200 in a satisfactory manner, while the time window of 18 hours is too large for such a number (vehicle occupancy was too low), hence the target number of 200 passengers and a two-hour time window were assumed for detailed tests. As part of the research work, tests were also carried out to check the impact of selected objective functions on the results obtained by individual optimization models. Three variants of models have been prepared, differing in the way vehicles move between bus stops: *General*, *Sections*, and *Routes* (see Section Levels of routing flexibility).

### Basic model

This subsection contains the results obtained by the basic constrained optimization model for two datasets. Tables 2 and 3 present the values of the parameters describing the results obtained by the basic model for three different variants (*General*, *Sections*, and *Routes*). The tables contain the following parameters: *t* – travel time, $T_n$ – number of tardy passengers, $T_\textrm{min}$ – minimum tardiness, $T_\textrm{avg}$ – average tardiness (4), $T_\textrm{max}$ – maximum tardiness (5a), $V_\textrm{avg}$ – average vehicle load. The effectiveness of a given model variant is best reflected in the coefficients related to passenger tardiness ($T_n$, $T_\textrm{min}$, $T_\textrm{avg}$, and $T_\textrm{max}$) because the method aims to select travel times so that passengers reach their designated destinations no later than at the declared time.

**Table 2:** The results of the basic model obtained for Dataset I.

	*t*[*s*]	$T_n$	$T_\textrm{min} [s]$	$T_\textrm{avg} [s]$	$T_\textrm{max} [s]$	$V_\textrm{avg} [\%]$
*General*	16296	140	0	2618	8408	45.56
*Sections*	10733	61	0	373.4	5017	31.77
*Routes*	9568	0	0	0	0	36.01

**Table 3:** The results of the basic model obtained for Dataset II.

	*t*[*s*]	$T_n$	$T_\textrm{min} [s]$	$T_\textrm{avg} [s]$	$T_\textrm{max} [s]$	$V_\textrm{avg} [\%]$
*General*	19707	160	0	4295	15388	43.29
*Sections*	11308	83	0	689.3	6161	30.04
*Routes*	10188	89	0	760.2	4887	36.07

As expected, the models perform much better in the case of Dataset I. Comparing the results obtained by the corresponding variants, it can be observed that the values of some parameters for Dataset I are even half those of Dataset II (e.g. $T_\textrm{avg}$). Moreover, for Dataset I, the algorithm (the *Routes* variant) found an ideal solution (none of the passengers were late). Analyzing the results achieved by the individual versions of the model, it can be seen that the *General* variant achieved the worst results. This is because in this variant the vehicle could move between any bus stops, which significantly increased the complexity of the problem. Already at the initial stage of work, it was noticed that this is why the model requires significant computation time and even after a very long time it is not able to find a solution with small passenger tardiness. For this reason, solutions were proposed to reduce the complexity of the problem (the *Sections* and *Routes* variants). For the aforementioned variants, a multiple reduction in the average tardy time was obtained. Moreover, the model found satisfactory solutions much faster. For Dataset I, the *General* variant obtained an average delay time of $T_\textrm{avg}$ = 2618 s (43 min), the *Sections* variant 373 s (6 min), while *Routes* found an ideal solution (none of the passengers were late). For Dataset II, *General* obtained $T_\textrm{avg}$ = 4295 s (72 min), the variant *Sections* 689 s (11 min), and *Routes* 760 s (13 min). Regarding vehicle occupancy (parameter $V_\textrm{avg}$), the value of this parameter is characteristic for individual variants and is the highest for the *General* ($V_\textrm{avg}$ = 43–45%). It might seem good that vehicle occupancy is high. In this case, however, it is most likely caused by non-optimal vehicle routes, which means that passengers have to stay in the vehicles for a long time. It is also worth paying attention to the total travel time of the vehicles. For the *General* variant, it is much longer than for the other variants. The high value of this parameter is also caused by non-optimal routes, which means that vehicles have to drive much longer than in the case of the other variants, which also results in significantly greater late of passengers.

**Fig. 2:**
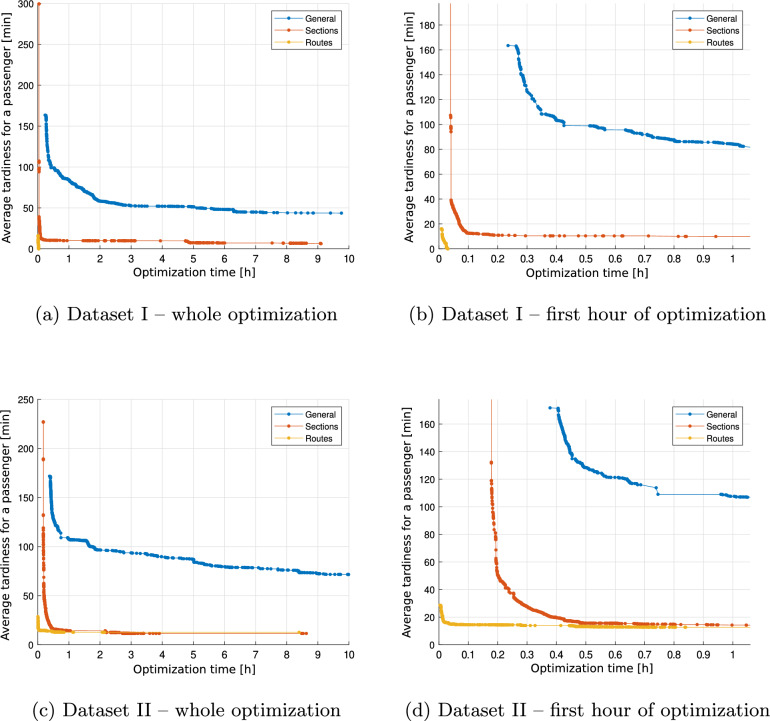
Changing the value of the objective function during the execution of the optimization basic model.

Figure 2 presents graphs of the change in the value of the objective function during the operation of the optimization method. Graphs (a) and (c) show 10 hours of the algorithm’s operation, while (b) and (d) show the first hour of optimization. The graph shows that variations of *Sections* and *Routes* can very quickly (*Sections* in less than 0.5 hours, while *Routes* in less than 0.1 hours) reduce to the value of average tardiness per passenger $T_\textrm{avg}$ below 20 minutes. Despite the much longer operation time, the *General* algorithm is not able to achieve delay values at this level. This variant is continually improving its solution, but it is a very slow process.

### Model with extension elements

The subsection presents the results obtained by the extended model of constrained optimization obtained for two sets of test data. Tables 4 and 5 present the parameters values describing the results obtained by the extended model for three different variants (*General*, *Sections*, and *Routes*). The results obtained present relationships similar to those in the case of the basic variant, but the values of individual parameters are higher. This is due to the fact that adding additional requirements complicates the problem, which affects the complexity of the model, and thus the obtained results.

**Table 4:** The results of the extended model obtained for Dataset I.

	*t*[*s*]	$T_n$	$T_\textrm{min} [s]$	$T_\textrm{avg} [s]$	$T_\textrm{max} [s]$	$V_\textrm{avg} [\%]$
*General*	17660	138	0	2450	8929	36.88
*Sections*	10338	49	0	383.6	4894	34.14
*Routes*	9890	0	0	0	0	40.66

**Table 5:** The results of the extended model obtained for Dataset II.

	*t*[*s*]	$T_n$	$T_\textrm{min} [s]$	$T_\textrm{avg} [s]$	$T_\textrm{max} [s]$	$V_\textrm{avg} [\%]$
*General*	20019	173	0	4687	14681	42.51
*Sections*	11426	105	0	1225	9402	26.25
*Routes*	10861	93	0	910.6	6778	26.62

**Fig. 3:**
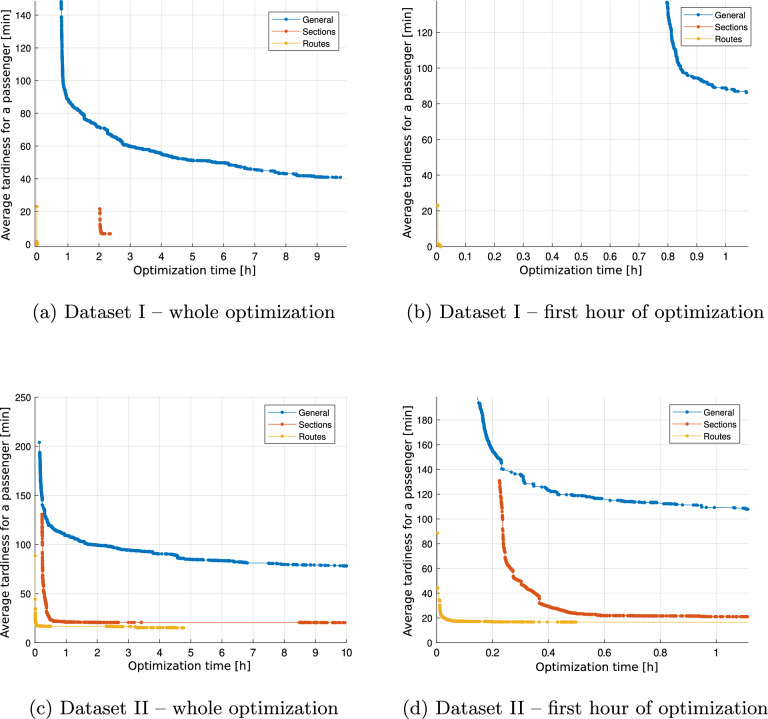
Changing the value of the objective function during the execution of the optimization method with extensions.

Figure 3 presents graphs of the change in the value of the objective function during the operation of the optimization method in the extended variant. Graphs (a) and (c) show 10 hours of the algorithm’s operation, while (b) and (d) the first hour of optimization. The courses obtained are analogous to those for the basic variant.

### Detailed results for the selected case

**Fig. 4:**
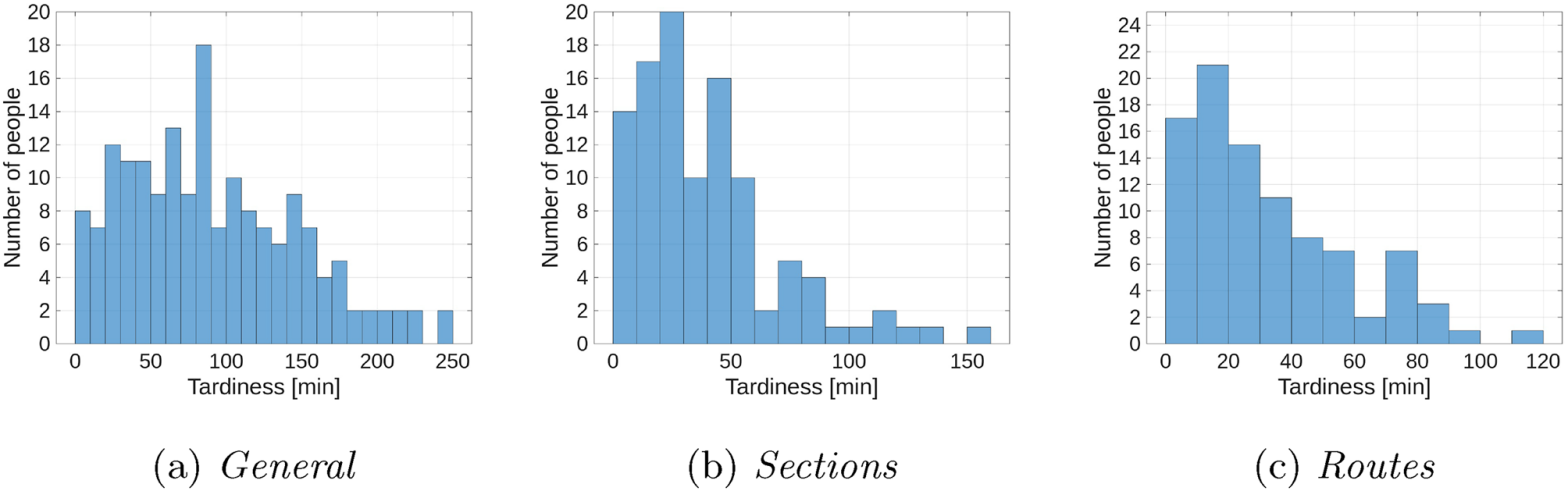
Histogram of passenger tardiness for the extended model and Dataset II.

The subsection presents detailed results for the extended model and Dataset II. This combination was chosen because its assumptions are the most realistic. Figure 4 shows a histogram of passenger tardiness. The graphs for the *Sections* (b) and *Routes* (c) variants are similar, which confirms the results discussed earlier in the form of tables. The tardiness for the *General* (a) variant is significantly greater than for *Sections* (b) and *Routes* (c).

**Fig. 5:**
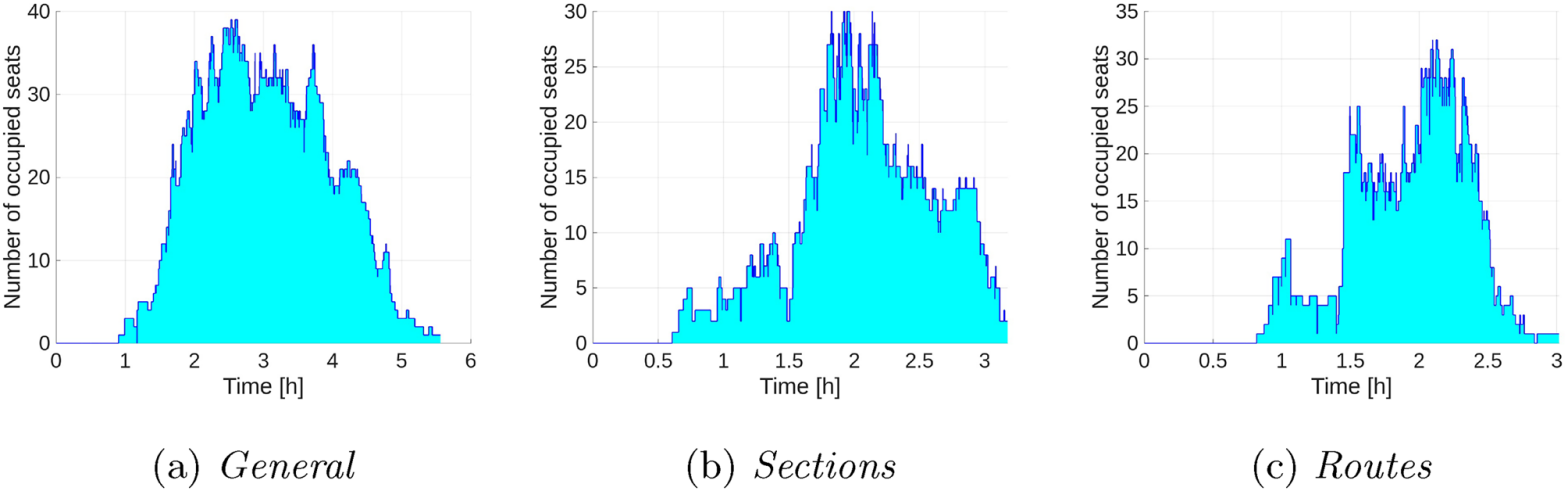
Total number of passengers per vehicle for the extended model and Dataset II.

Figure 5 illustrates the number of passengers traveling in all vehicles at a given time. Again, for the *General* variant (a) we can observe a long total time and a large number of passengers in the vehicles. The graphs for the *Sections* (b) and *Routes* (c) variants are similar in terms of both time and the number of passengers traveling in the vehicles.

**Fig. 6:**
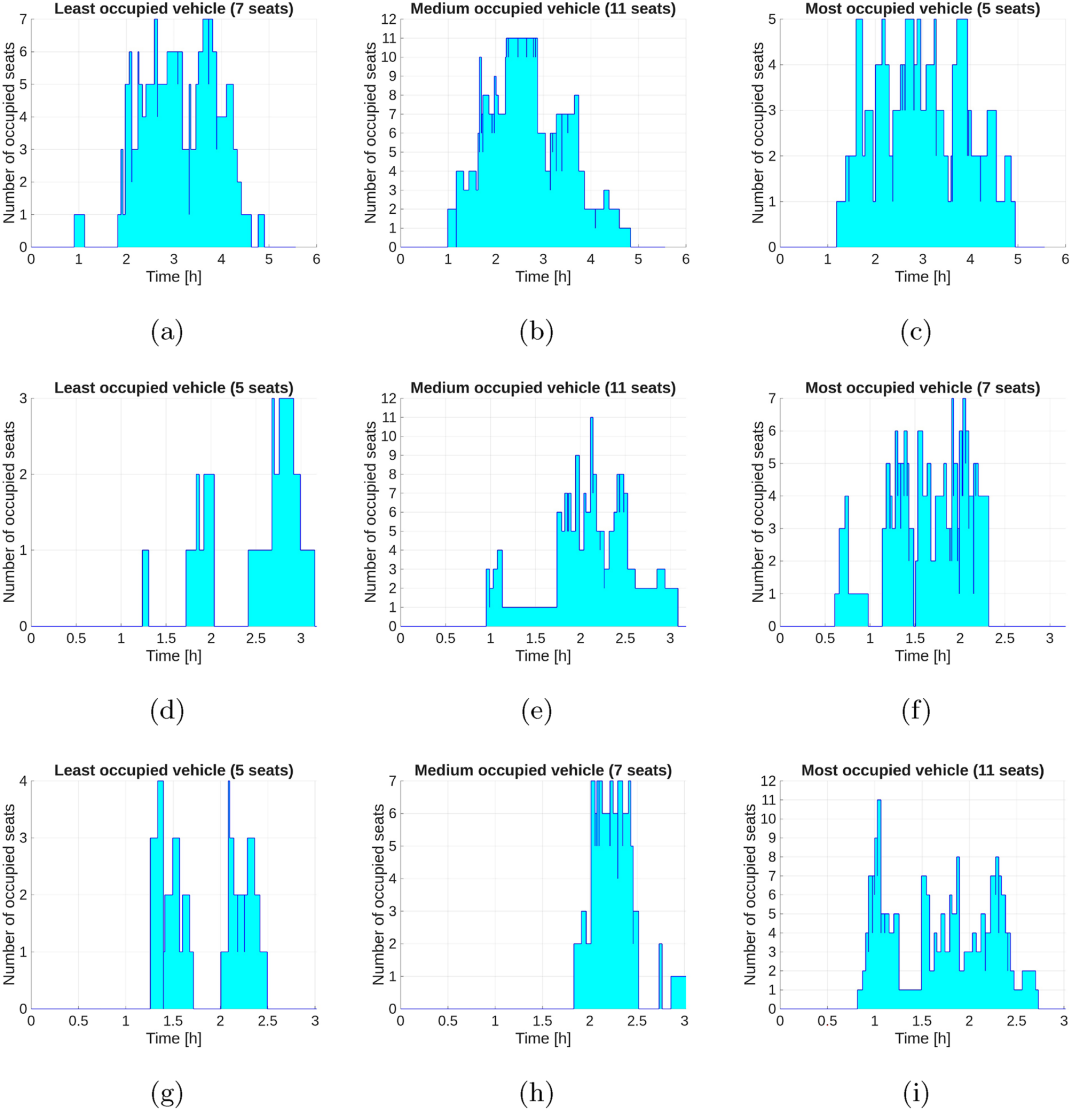
Vehicle load for data set II; (a) *General* – least occupied vehicle, (b) *General* – medium occupied vehicle, (c) *General* – most occupied vehicle, (d) *Sections* – least occupied vehicle, (e) *Sections* – medium occupied vehicle, (f) *Sections* – most occupied vehicle, (g) *Routes* – least occupied vehicle, (h) *Routes* – medium occupied vehicle, (i) *Routes* – most occupied vehicle.

**Fig. 7:**
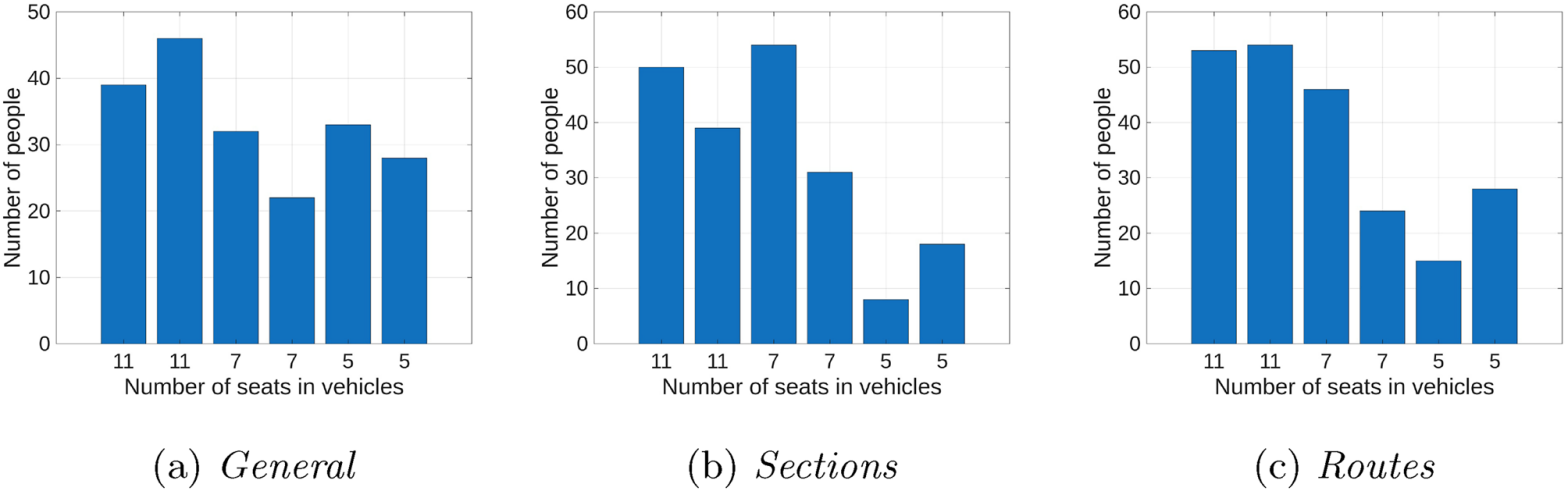
Number of passengers transported by individual vehicles for Dataset II.

The graphs in Fig. 6 show the load of the least, medium and most loaded vehicles, while Fig. 7 shows the number of passengers carried by individual vehicles. As can be seen, vehicles are loaded very differently. By analyzing the graphs discussed, you can draw conclusions regarding the optimal number of vehicles for a given number of passengers. If there are too few vehicles, they will be well loaded, but unfortunately the tardiness of passengers will be large, while if there are too many vehicles, it will be easier to find a solution, but some vehicles will drive with a small number of passengers. Six vehicles were used in the tests conducted and this was the optimal number for the prepared test data.

### Discussion of vehicle charging results

**Fig. 8:**
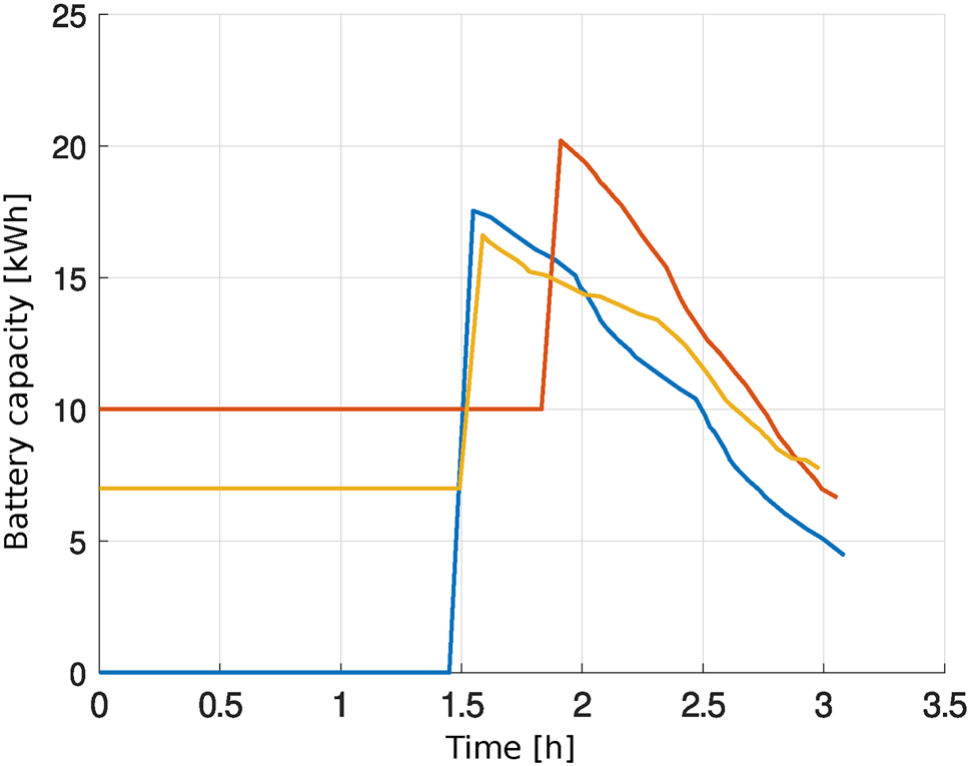
Change in battery charge of electric vehicles.

The developed model also takes into account the process of discharging and charging batteries of electric vehicles that carry passengers. Figure 8 shows the change in the battery state of electric vehicles over time for the *Routes* variant. The experiment used three electric vehicles with different initial battery charge states. The developed method selected the location, start time, and charging time so that the vehicle battery charge level did not drop below a certain critical value (in the experiment it was 0, but any other value can be set). The graph shows an increase in the battery charge level, which is the result of charging, and a decrease in the battery level as a result of the vehicle driving between stops.

## Evaluation of selected objective functions

**Fig. 9:**
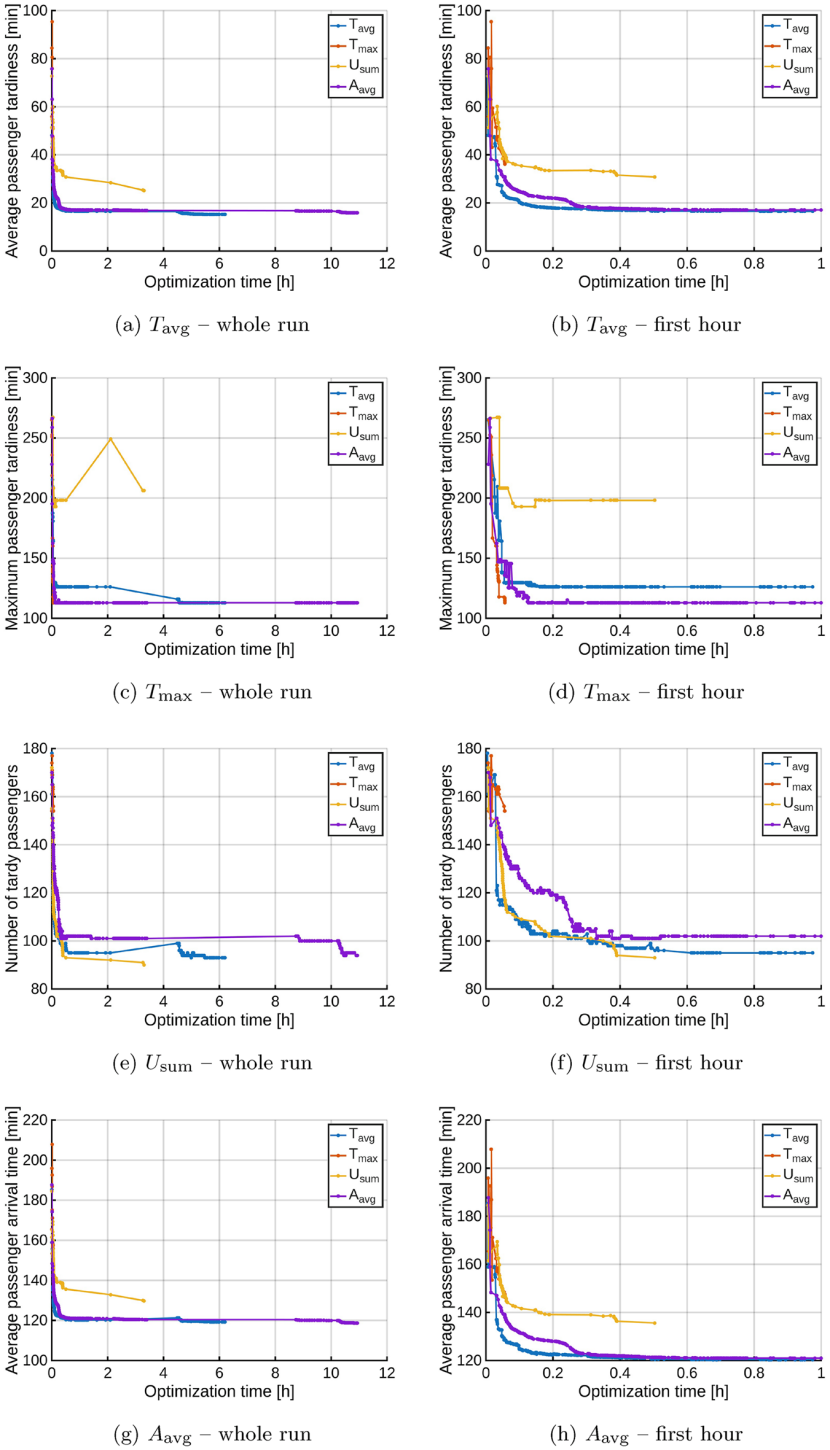
The impact of different objective functions on the results achieved.

Each of the comparative graphs in Fig. 9 shows the changes in the value of one of the objective functions (in order: $T_\textrm{avg}$, $T_\textrm{max}$, $U_\textrm{sum}$ and $A_\textrm{avg}$), when the active optimization criterion is successively all of these functions. As expected, the value of the function selected as the criterion is minimized most effectively, or at least not worse than that of other functions. The following conclusions can be drawn from the comparison:

1. The functions $T_\textrm{avg}$ and $A_\textrm{avg}$ change almost simultaneously or at least obtain very similar values after a sufficiently long time, regardless of the criterion used. This can be explained by the similar construction of the expressions defining these functions, which are linear with respect to $\sum _{t\in T}a_t$, unless the condition $a_t<\omega _t$ is met.
2. The optimization based on $T_\textrm{max}$ turns out to be practically useless, because this objective function also obtains the same minimum value for the criteria $T_\textrm{avg}$ and $A_\textrm{avg}$ (Fig. 9c, 9d). This means that using $T_\textrm{avg}$ or $A_\textrm{avg}$ will give the same tardiness to the most tardy passenger as using $T_\textrm{max}$; and, additionally, the tardiness of other passengers will also be minimized, which does not occur for $T_\textrm{max}$ (Fig. 9a, 9b, 9g, 9h).
3. The objective function $U_\textrm{sum}$ is clearly in a weak correlation with the other considered ones. When the number of tardy passengers is minimized, both the total and maximum tardiness increase significantly, which leads us to consider the communication plan to be globally less efficient. Additionally, if the criterion $T_\textrm{avg}$ or $A_\textrm{avg}$ is applied, the number of tardy passengers is minimized almost as effectively as with $U_\textrm{sum}$ (Fig. 9e, 9f).

The results confirm that the choice of $T_\textrm{avg}$ as the basic objective function used for most experiments is justified. Additionally, it turns out that the function $A_\textrm{avg}$ is also useful in practice, as it effectively enforces the fastest possible arrival times of passengers, achievable in the communication system, even if the passengers did not specify their desired destinations. The other two objective functions $T_\textrm{max}$ and $U_\textrm{sum}$ are less useful.

## Discussion

The proposed DRT optimization model has been evaluated using realistic data based on the location of bus stops in Rzeszow and numerical parameters (Table 1). The objective function in the form of average passenger tardiness is a measure of practical operational performance and service quality.

In each of the experiments performed, the *Routes* flexibility level is the best, both in terms of the optimization speed and the final value of the objective function. The *Sections* level performs only slightly worse in minimizing the objective function, whereas the *General* level is definitely the weakest. This is also clearly visible in the example of seat occupancy (Fig. 5), where the area under the graph in the case of the *General* level is the largest, which means that passengers spend on average the most time in vehicles and therefore the travel plan is the least efficient. This result is somewhat consistent with predictions of established traffic-flow principles stating that the level of service (LOS) in public transport systems is not improved by reducing routing constraints. However, this result can also be seen as a limitation of the algorithm used when searching a solution space that is too large. Further research is needed, in particular on the basis of other optimization algorithms.

The purpose of this work is to create an efficient, complete, and realistic optimization model for a DTR system. To achieve this, the model has a two-level formulation, with the second level containing more detailed elements and constraints (e.g. those related to electric vehicles). This level can be modified and adapted to a specific case, increasing flexibility and practical usefulness.

This work focuses on DRT optimization and therefore does not directly address other aspects of this field. However, integration with these aspects and the use of their results are possible and anticipated in future work. The most important of these include:

1. More realistic modeling of road traffic, taking into account roadway capacity, traffic flow stability, or LOS theory.
2. Application of more precise models of vehicle automation and traffic control systems^30^.
3. Incorporation of AVs and ACC vehicles in the optimization model^29^.
4. Extending the developed model and optimization algorithm to a full-scale MaaS system^12^.
5. Adaptation of the optimization model to multimodal transport.
6. Application of passenger opinion and behavior analysis methods, e.g., based on the Technology Acceptance Model (TAM) and Random Utility Maximization (RUM)^12^ approaches, to better adjust the structure and parameters of the optimization model.

## Conclusions

The aim of the work was to present and verify the general two-level framework for DRT. The first level includes the basic features essential for every DRT system. The second level takes into account the management of electric vehicles, the different initial states of vehicles, as well as special passenger requests (e.g., a dedicated space for disabled people, a vending machine with drinks, availability of a Wi-Fi network, etc.). Three variants of models have been prepared, differing in the way vehicles move between bus stops: *General*, *Sections*, and *Routes*. During experiments, the developed model was thoroughly tested; specifically, its effectiveness in the basic version and with additional extensions was evaluated. We also analyzed in detail the optimization times of different objective functions, the delays of passengers, vehicle loads, and changes in battery charge. The experiments conducted confirmed the usefulness of the proposed approach. The research yielded the following three key results:

1. A two-level universal optimization model for the DRT problem was developed, which served as a basis for future implementations.
2. The effectiveness of three levels of routing flexibility (G, S, R) was compared and it was observed that the most restrictive variant provided, somewhat unexpectedly, the best results, which can be associated with a reduction in the size of the solution space.
3. A complete set of optimization results was obtained for the test data, providing a starting point for comparisons with other methods.

It should also be mentioned that the proposed method has some limitations. Due to the high complexity of the problem, some simplifications were made, for instance:

- travel times are fixed for any pair of stops and do not take into account, for example, the time of day;
- a static plan of passenger transfers is adopted, minimizing their number and travel time, but not taking into account waiting at stops;
- a constant vehicle battery charging power is assumed that depends only on the charger at a given bus stop. In addition, the battery discharge is simply proportional to the distance.

Such elements can be extended and better adapted to real-world conditions in future work.

The proposed optimization model is linear, and dynamic indexing can be replaced with expressions based on binary variables using standard techniques. Hence, the mixed-integer linear programming (MILP) implementation of the model is planned in the future to compare its performance with the current constraint programming version. Future work also involves the use of promising metaheuristic optimization methods, for instance, tabu search or simulated annealing, as well as those inspired by nature such as ant colony, bee colony, or particle swarm optimization.

## Supplementary Information

Supplementary Information.

## Data Availability

All data generated or analyzed during this study are included in this published article and its supplementary information files.

## References

[1] Franco, P., Johnston, R. & McCormick, E. Demand responsive transport: Generation of activity patterns from mobile phone network data to support the operation of new mobility services. *Transp. Res. A Policy Pract.* **131**, 244–266. 10.1016/j.tra.2019.09.038 (2020).

[2] Guo, R., Zhang, W., Guan, W. & Ran, B. Time-dependent urban customized bus routing with path flexibility. *IEEE Trans. Intell. Transp. Syst.* **22**(4), 2381–2390. 10.1109/tits.2020.3019373 (2021).

[3] Wu, Y., Yuan, Z., Xiao, Q. & Yang, D. Customized bus scheme design of large transport terminals with jointly optimization of departure time, vehicle allocation and routing. *IET Intell. Transp. Syst.* **17**(1), 85–101. 10.1049/itr2.12240 (2022).

[4] Xiong, J., Chen, B., Li, X., He, Z. & Chen, Y. Demand responsive service-based optimization on flexible routes and departure time of community shuttles. *Sustainability* **12**(3), 897. 10.3390/su12030897 (2020).

[5] Inturri, G. et al. Taxi vs. demand responsive shared transport systems: An agent-based simulation approach. *Transp. Policy.* **103**, 116–126. 10.1016/j.tranpol.2021.01.002 (2021).

[6] Ho, S. C. et al. A survey of dial-a-ride problems: Literature review and recent developments. *Transp. Res. B Methodol.* **111**, 395–421. 10.1016/j.trb.2018.02.001 (2018).

[7] Ma, T.-Y. Optimal Battery Replenishment and Vehicle-charger Assignment for Dynamic Electric Demand Responsive Transport Service, pp. 134–134 (2021). 31st European Conference on Operational Research ; Conference date: 11-07-2021 Through 14-07-2021. https://euro2021athens.com/

[8] Shu, W. & Li, Y. A novel demand-responsive customized bus based on improved ant colony optimization and clustering algorithms. *IEEE Trans. Intell. Transp. Syst.* **24**(8), 8492–8506. 10.1109/tits.2022.3145655 (2023).

[9] Vansteenwegen, P. et al. A survey on demand-responsive public bus systems. *Transp. Res. C Emerg. Technol.* **137**, 103573. 10.1016/j.trc.2022.103573 (2022).

[10] Guo, R., Guan, W., Zhang, W., Meng, F. & Zhang, Z. Customized bus routing problem with time window restrictions: Model and case study. *Transportmetrica A Transp. Sci.* **15**(2), 1804–1824. 10.1080/23249935.2019.1644566 (2019).

[11] Liu, K., Liu, J. & Zhang, J. Heuristic approach for the multiobjective optimization of the customized bus scheduling problem. *IET. Intell. Transp. Syst.* **16**(3), 277–291. 10.1049/itr2.12131 (2021).

[12] Meloni, I. et al. Mobility as a service: Insights from pilot studies across different Italian settings. *Transp. Eng.* **18**, 100294. 10.1016/j.treng.2024.100294 (2024).

[13] Coutinho, F. M. et al. Impacts of replacing a fixed public transport line by a demand responsive transport system: Case study of a rural area in Amsterdam. *Res. Transp. Econ.* **83**, 100910. 10.1016/j.retrec.2020.100910 (2020).

[14] Schlüter, J., Bossert, A., Rössy, P. & Kersting, M. Impact assessment of autonomous demand responsive transport as a link between urban and rural areas. *Res. Transp. Bus. Manag.* **39**, 100613. 10.1016/j.rtbm.2020.100613 (2021).

[15] Dytckov, S., Persson, J. A., Lorig, F. & Davidsson, P. Potential benefits of demand responsive transport in rural areas: A simulation study in Lolland, Denmark. *Sustainability* **14**(6), 3252. 10.3390/su14063252 (2022).

[16] Martí, P., Jordán, J., Prieta, F. & Julian, V. Optimization of rural demand-responsive transportation through transfer point allocation. *Electronics* **12**(22), 4684. 10.3390/electronics12224684 (2023).

[17] Bruzzone, F., Scorrano, M. & Nocera, S. The combination of e-bike-sharing and demand-responsive transport systems in rural areas: A case study of Velenje. *Res. Transp. Bus. Manag.* **40**, 100570. 10.1016/j.rtbm.2020.100570 (2021). 38620580 10.1016/j.rtbm.2020.100570 PMC7522006

[18] Lakatos, A., Tóth, J. & Mándoki, P. Demand responsive transport service of ‘dead-end villages’ in interurban traffic. *Sustainability* **12**(9), 3820. 10.3390/su12093820 (2020).

[19] Costa, P. C., Cunha, C. B. & Arbex, R. O. A simulation-optimization model for analyzing a demand responsive transit system for last-mile transportation: A case study in São Paulo, Brazil. *Case Stud. Transp. Policy.* **9**(4), 1707–1714. 10.1016/j.cstp.2021.06.019 (2021).

[20] Song, C., Wang, H., Chen, L. & Niu, X. An optimized two-phase demand-responsive transit scheduling model considering dynamic demand. *IET Intell. Transp. Syst.* **18**(5), 853–871. 10.1049/itr2.12473 (2023).

[21] Calabrò, G. et al. An exploratory study using an agent-based model. *J. Adv. Transp.* **2022**, 1–20. 10.1155/2022/8382754 (2022).

[22] Zhao, J., Sun, S. & Cats, O. Joint optimisation of regular and demand-responsive transit services. *Transportmetrica A. Transp. Sci.*10.1080/23249935.2021.1987580 (2021).

[23] Ma, T.-Y. & Fang, Y. Survey of charging management and infrastructure planning for electrified demand-responsive transport systems: Methodologies and recent developments. *Eur. Transp. Res. Rev.*10.1186/s12544-022-00560-3 (2022).

[24] Masson, R., Lehuédé, F. & Péton, O. The dial-a-ride problem with transfers. *Comput. Oper. Res.* **41**, 12–23. 10.1016/j.cor.2013.07.020 (2014).

[25] Lee, A. & Savelsbergh, M. An extended demand responsive connector. *EURO J. Transp. Logist.* **6**(1), 25–50. 10.1007/s13676-014-0060-6 (2017).

[26] Liang, S. et al. Optimal control to improve reliability of demand responsive transport priority at signalized intersections considering the stochastic process. *Reliab. Eng. Syst. Saf.* **218**, 108192. 10.1016/j.ress.2021.108192 (2022).

[27] Papanikolaou, A., Basbas, S., Mintsis, G. & Taxiltaris, C. A methodological framework for assessing the success of Demand Responsive Transport (DRT) services. *Transp. Res. Procedia.* **24**(2016), 393–400. 10.1016/j.trpro.2017.05.095 (2017).

[28] Wang, H., Li, J., Wang, P., Teng, J. & Loo, B. P. Y. Adaptability analysis methods of demand responsive transit: A review and future directions. *Transp. Rev.* **43**(4), 676–697. 10.1080/01441647.2023.2165574 (2023).

[29] Mohammed, D. & Horváth, B. Steady-speed traffic capacity analysis for autonomous and human-driven vehicles. *Applied Sciences* **14**(1). 10.3390/app14010337 (2024).

[30] Mohammed, D., Nagy, V., Jagicza, M., Józsa, D. & Horváth, B. Efficiency of adaptive cruise control in commercial vehicles. *Pollack Period.* **19**(2), 1–7. 10.1556/606.2024.00970 (2024).

[31] Davison, L., Enoch, M., Ryley, T., Quddus, M. & Wang, C. A survey of demand responsive transport in great britain. *Transp. Policy* **31**, 47–54. 10.1016/j.tranpol.2013.11.004 (2014).

[32] Molenbruch, Y., Braekers, K. & Caris, A. Typology and literature review for dial-a-ride problems. *Ann. Oper. Res.* **259**(1–2), 295–325. 10.1007/s10479-017-2525-0 (2017).

[33] Filippi, C., Guastaroba, G., Peirano, L. & Speranza, M. G. Trends in passenger transport optimisation. *Int. Trans. Oper. Res.* **30**(6), 3057–3086. 10.1111/itor.13300 (2023).

[34] Huang, D., Gu, Y., Wang, S., Liu, Z. & Zhang, W. A two-phase optimization model for the demand-responsive customized bus network design. *Transp. Res. C Emerg. Technol.* **111**, 1–21. 10.1016/j.trc.2019.12.004 (2020).

[35] Laborie, P., Rogerie, J., Shaw, P. & Vilím, P. Ibm ilog cp optimizer for scheduling. *Constraints* **23**, 210–250. 10.1007/s10601-018-9281-x (2018).

